# Comparison of Statistical Population Reconstruction Using Full and Pooled Adult Age-Class Data

**DOI:** 10.1371/journal.pone.0033910

**Published:** 2012-03-28

**Authors:** John R. Skalski, Joshua J. Millspaugh, Michael V. Clawson

**Affiliations:** 1 School of Aquatic and Fishery Sciences, University of Washington, Seattle, Washington, United States of America; 2 Department of Fisheries and Wildlife Sciences, University of Missouri, Columbia, Missouri, United States of America; 3 School of Forest Resources, University of Washington, Seattle, Washington, United States of America; University of Leeds, United Kingdom

## Abstract

**Background:**

Age-at-harvest data are among the most commonly collected, yet neglected, demographic data gathered by wildlife agencies. Statistical population construction techniques can use this information to estimate the abundance of wild populations over wide geographic areas and concurrently estimate recruitment, harvest, and natural survival rates. Although current reconstruction techniques use full age-class data (0.5, 1.5, 2.5, 3.5, … years), it is not always possible to determine an animal's age due to inaccuracy of the methods, expense, and logistics of sample collection. The ability to inventory wild populations would be greatly expanded if pooled adult age-class data (e.g., 0.5, 1.5, 2.5+ years) could be successfully used in statistical population reconstruction.

**Methodology/Principal Findings:**

We investigated the performance of statistical population reconstruction models developed to analyze full age-class and pooled adult age-class data. We performed Monte Carlo simulations using a stochastic version of a Leslie matrix model, which generated data over a wide range of abundance levels, harvest rates, and natural survival probabilities, representing medium-to-big game species. Results of full age-class and pooled adult age-class population reconstructions were compared for accuracy and precision. No discernible difference in accuracy was detected, but precision was slightly reduced when using the pooled adult age-class reconstruction. On average, the coefficient of variation 
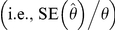
 increased by 0.059 when the adult age-class data were pooled prior to analyses. The analyses and maximum likelihood model for pooled adult age-class reconstruction are illustrated for a black-tailed deer (*Odocoileus hemionus*) population in Washington State.

**Conclusions/Significance:**

Inventorying wild populations is one of the greatest challenges of wildlife agencies. These new statistical population reconstruction models should expand the demographic capabilities of wildlife agencies that have already collected pooled adult age-class data or are seeking a cost-effective method for monitoring the status and trends of our wild resources.

## Introduction

The collection of age-at-harvest data is a routine activity of most state and provincial wildlife management agencies. For many agencies an assessment of annual harvest for big game is made using hunter check stations [1], [2]. In addition to total harvest, data on sex and age-at-harvest are routinely collected at mandatory check stations or obtained from hunters that use postage-paid envelopes to mail a tooth to the management agency (e.g., [3]). Not all harvested animals need to be aged, just a representative sample of the harvest. These harvest data are often the only wide-scale data available on an annual basis to assess population status and trends for these species, efficacy of harvest regulations, and response of these populations to management activities. Although such data can be collected relatively inexpensively, management agencies must make decisions regarding the level of detail required necessary to meet their objectives. Managers need to determine whether full age-class data should be collected (e.g., 0.5, 1.5, 2.5, 3.5…) or whether pooling animals by age category (e.g., 0.5, 1.5, 2.5+) is sufficient.

For harvested big game species, there are multiple options for aging animals, but each has distinct benefits and drawbacks. For many mammals, counts of cementum annuli [4] often provide the most accurate estimate of age [5]. However, the process of collecting, sectioning and counting cementum annuli can be expensive and time consuming when applied across broad geographic regions. Also, counts of cementum annuli are not error free [6], [7], [8]. Many alternative, less precise, methods have been used to estimate age of harvested animals. For ungulate populations, age determination can be based on tooth eruption and wear [9], [10]. This inexpensive aging technique is often accurate for individuals ≤2.5 or ≤3.5 years of age, depending on species ([11]:190–194), but accuracy can be as low as 16% for elk (*Cervus elaphus*) ≥5 years of age [5]. For this reason, most researchers are only comfortable with assigning animals, such as deer, to age categories of fawn, yearling, and adult [12]. For other species, including some carnivores, investigators have used tooth pulp cavity metrics to assign age categories. River otters (*Lutra canadensis*) have been classified to juvenile and adult stages using pulp cavity width through examination of radiographs [13]. A similar method has been used to age male and female fishers to age classes 0, 1, 2, and 3+ [14]. Therefore, although it is difficult to accurately assign full age-class data, it might still be possible to pool animals into biologically relevant stages or age categories. An additional advantage to pooling might be reduced cost compared to precise age determination [14], which may be especially important when considering state-wide harvest assessments. However, biologists need to know how pooling age-class data into age categories rather than collecting full age-class data would affect the intended demographic analyses.

Harvest data commonly are analyzed using population reconstruction methods [15]. Although still commonly used by state management agencies, many of the early deterministic reconstruction methods have substantial bias and make unrealistic assumptions [16]. In contrast, statistical population reconstruction techniques have lower bias, require a more realistic set of assumptions, provide a flexible framework which can include auxiliary data [17], [15], [18], and allow simultaneous estimation of multiple demographic parameters such as natural survival rates and abundance [15] as well as confidence intervals for these parameters. However, statistical population reconstruction methods have typically relied on full age-class information to reconstruct cohort and annual abundances [17], [15]. There would be practical, economical, and logistical benefits if age category data could be used in population reconstruction analysis. However, it is unknown whether pooled age-class data, which maintain less resolution than full age-class data, can support reliable estimation of demographic parameters.

In this paper, we use simulation studies to compare statistical population reconstruction results using full age-class data [18] and pooled age categories of 0.5, 1.5, and 2.5+ years over a wide range of abundance levels, natural survival probabilities, and harvest rates. Our objective is to assess whether reliable abundance estimates can be obtained by pooling age-class data. We illustrate these techniques using age-at-harvest data from a Columbian black-tailed deer (*Odocoileus hemionus columbianus*) population [18].

## Methods

### Simulation Study

A Monte Carlo simulation study was used to determine the accuracy and precision of population reconstructions based on pooled adult age-class data and compare their performance to full age-class analyses. A stochastic version of a Leslie matrix model was used to generate age-at-harvest data for populations with different levels of total abundance, natural survival rates, and harvest rates. Recruitment levels were adjusted to produce populations with stationary abundance in expectation but fluctuated as a result of random recruitment and survival processes. Recruitment was generated using a Poisson process and natural survival and harvest generated as binomial processes.

In each simulation, 20 years of data were generated to establish demographic trends with years 21–44 used in the population reconstructions. Full age-class data were generated and used in standard population reconstruction models [18]. The same data were also reanalyzed after pooling the adult age-at-harvest data (i.e., 2.5+ year olds) using the pooled adult reconstruction model described in the next section. A total of 10,000 simulations were performed per demographic scenario.

Demographic scenarios were performed to represent a wide range of big game scenarios. Two levels of population abundance were simulated, low abundance of 1,000–3,000 animals and high abundance of 10,000–30,000 animals. Natural survival probabilities were simulated at 0.60, 0.75, or 0.90 and harvest rates at 0.10, 0.175, or 0.25. To minimize the number of scenarios investigated, survival and harvest rates were assumed constant across all age classes. Although auxiliary data (i.e., radiotelemetry, independent abundance estimates, etc.) can help with the accuracy and precision of population reconstructions, the simulations were performed without such data to mimic the 24 years of black-tailed deer data presented in the example below.

Accuracy of the population reconstructions was examined by calculating relative bias of the annual abundance estimates for each scenario, defined as
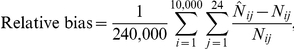
where *N_ij_* is the true abundance in the *j*th year (*j* = 1, …, 24) of the *i*th simulation (*i* = 1, …, 10,000) and where 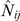 is the associated estimate. Sampling precision was estimated independent of the model for each scenario and expressed in terms of an average coefficient of variation (CV) where
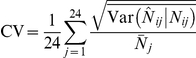
based on the expected value of the variance components
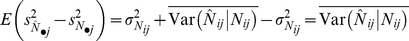
where 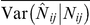 is average measurement error, 
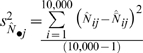
 is the empirical variance among the estimates of abundance, and where 
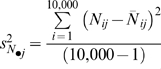
 is the empirical variance among simulated abundance values. A total of 10,000 simulations per scenario were used to obtain precise estimates of 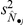 and 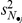 as a means of obtaining model-independent estimates of average measurement error.

### Expository Example: Black-tailed Deer

#### Study area

We used harvest data for Columbian black-tailed deer from the 22,079-ha King Creek block of Kapowsin Tree Farm, Pierce County, Washington, to illustrate the pooled age reconstruction methods [18]. A detailed description of the study area is available [19]. Controlled access to the area permitted a complete tally of all animals harvested, their ages, and hunter effort.

#### Likelihood model

From 1979–2000, all harvested female deer within the study area, were aged to a specific year from cementum annuli [19]. With full age-class data, population reconstruction is based on estimating the annual abundance levels of the separate cohorts constituting the population [17], [15], [18]. The full age-class model we used was reported previously [18].

We reanalyzed the Columbia black-tailed deer data set after pooling ages 2.5+ years into one category. When harvest data from older age classes are pooled, information about cohort structure is retained only for the youngest age classes (i.e., 0.5 and 1.5). Nevertheless, this truncated cohort structure of the data can be used to help structure the population reconstruction. As with the full age-class model, the statistical model for the population reconstruction using pooled data is based on a joint likelihood model of the form
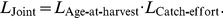
(1)Because of pooling the harvest data from age classes in the 2+ category, the structure of the age-at-harvest likelihood necessarily changes. With pooling, the likelihood takes the form
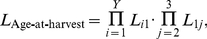
(2)where 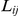 is the likelihood describing the age-at-harvest data for the cohort entering the study in year 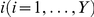 at age category 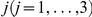. Let


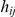
 = number of animals harvested in year *i* at age category *j*;


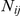
 = deer abundance in year *i* at age category *j*;


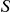
 = natural survival probability;


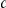
 = vulnerability coefficient that translates hunter effort to harvest probability;


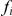
 = hunter effort in year *i*.

A previous analysis found separate vulnerability coefficients were needed for age class 0.5 (i.e., *c*_0.5_) and older animals in age classes 1.5 and above (i.e., *c*_1.5+_) in reconstruction of this population [15]. This same parameterization was used in this comparison of full and pooled adult age-class data. A common, annual natural survival probability was found to be adequate for this population [15].

For the adults already present in the population in year 1 (i.e., *N*_13_), their likelihood contribution can be written as follows:
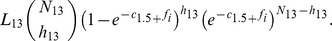
For the yearlings present in the population in year 1 (i.e., *N*_12_), their harvest in the first year and their harvest with other adults in the next year, as based on the conditional likelihood, was as follows:
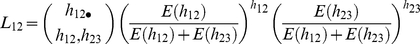
where
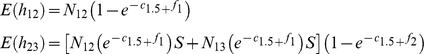
and where 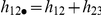. For the juveniles present in the first year (i.e., *N*_11_), the likelihood can be written as follows:
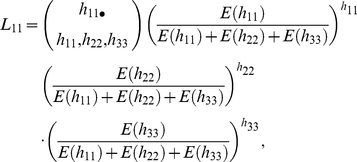
where
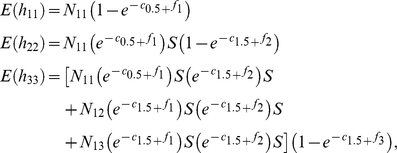



and where 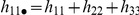. Construction of 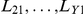 is analogous to that of 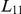 incremented for subsequent years. The likelihood contribution 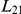 will include the expected values for the harvest counts 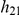, 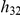, and 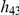. The expected value for 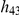 will include individuals from *N*_21_ that survive to be harvested as adults, plus adults that survive from the previous year that are subsequently harvested in year 3, and composed of animals from cohorts *N*_11_, *N*_12_, and *N*_13_.

The catch-effort likelihood is used to model the relationship between hunter effort and harvest rates
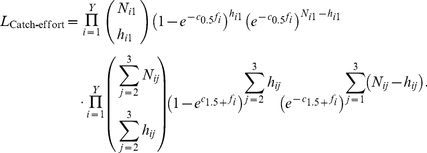
(3)


We fit a likelihood model to both the full and pooled data with a common natural survival probability and separate vulnerability coefficients (*c*) for the young-of-year and older females. We used the statistical software Program *USER* (University of Washington, http://www.cbr.washington.edu/paramest/user/) to solve for the maximum likelihood estimates. Initial abundance levels (i.e., 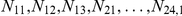) were estimated directly from the likelihood model while the remaining abundance values were calculated based on the invariance property of the maximum likelihood estimation, where
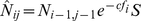
(4)Total annual abundance for each year was the sum of the estimated abundances for the three age cohorts for that year. We calculated standard errors from the inverse hessian, which was estimated numerically. The estimated standard errors were adjusted by a scale parameter estimated from a chi-square goodness-of-fit statistic [15]. We compared the full and pooled age class results by correlating the annual abundance estimates from the two approaches and by comparing the precision of the two techniques.

## Results

### Simulation Study

Fourteen different demographic scenarios were simulated 10,000 times each, with population abundance reconstructed over a 24-year period, using both the full age-class and pooled adult age-class information (Table 1). The relative bias for the full age-class analyses ranged from −0.0086 to +0.0198. The relative bias of the pooled adult age-class analyses had a similar range of −0.0093 to +0.0247. The average relative difference in abundance estimates between techniques range from −0.0110 to +0.0130, which suggests the two reconstruction methods can produce comparable results over the wide range of demographic conditions simulated.

**Table 1:** Simulation results* for pooled adult age-class and full age-class population reconstructions with an average percent bias of abundance estimates from the pooled age class analysis (*N_P_*), the relative bias for the full age-class analyses (*N_F_*), average relative difference between estimation techniques, and average coefficient of variation (CV) for the two approaches.

Simulation	Abundance	Survival	Harvest	(*N_F_* – *N*)/*N*	(*N_P_* – *N*)/*N*	(*N_P_* – *N_F_*)/*N*	CV_F_	CV_P_
1	L	H	H	−0.0086	−0.0093	−0.0007	0.137	0.146
2	L	M	H	0.0024	−0.0031	−0.0054	0.143	0.175
3	L	M	M	0.0019	0.0070	0.0051	0.122	0.183
4	L	M	L	0.0097	0.0214	0.0118	0.123	0.224
5	L	L	H	0.0046	−0.0061	−0.0108	0.168	0.185
6	L	L	M	0.0060	0.0044	−0.0016	0.155	0.250
7	L	L	L	0.0198	0.0247	0.0048	0.144	0.228
8	H	H	H	0.0081	0.0032	−0.0048	0.111	0.151
9	H	M	H	0.0113	0.0031	−0.0083	0.144	0.177
10	H	M	M	0.0096	0.0115	0.0019	0.137	0.209
11	H	M	L	0.0079	0.0209	0.0130	0.137	0.281
12	H	L	H	0.0129	0.0019	−0.0110	0.176	0.199
13	H	L	M	0.0133	0.0102	−0.0032	0.226	0.213
14	H	L	L	0.0120	0.0196	0.0076	0.170	0.293

* Simulations were conducted at two population levels (i.e., L = 1,000–3,000, H = 10,000–30,000), three levels of natural survival (i.e., H = 0.90, M = 0.75, L = 0.60), and three probabilities of harvest (H = 0.25, M = 0.10, L = 0.05).

Precision of the pooled adult age-class analyses was slightly less than that of the full age-class population reconstruction, on average. Across the simulations, the average CVs for pooled analyses ranged from 0.146 to 0.293. For the full age-class analyses, average CVs ranged from 0.111 to 0.226. There was, on average, a 0.059-point increase in expected CVs from the full to pooled simulations. These simulations therefore suggest there is no degradation in accuracy and a small decrease in precision, on average, when using pooled adult age-class data in population reconstructions. The decreased precision may be justified by the reduced costs of aging animals to only young-of-year, yearlings, and adults (2.5+ years in age).

### Expository Example: Black-Tailed Deer

**Figure 1:**
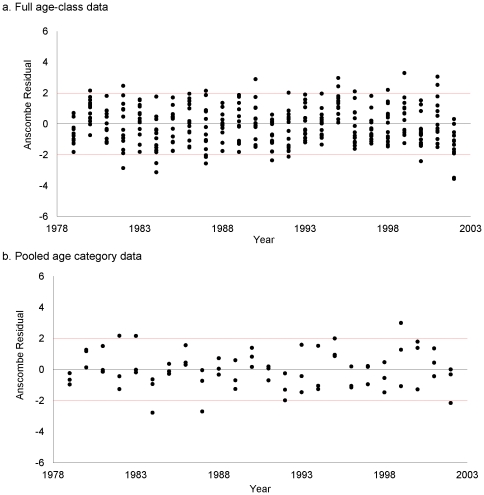
Standardized residuals [**23**] plotted by calendar year for the black-tailed deer population reconstruction using (a) full age-class data and (b) pooled age-class data (i.e., 0.5, 1.5, and 2.5+). a. Full age-class data. b. Pooled age category data.

All comparisons and diagnostics indicated little difference in the values and properties of the full and pooled adult age-class population reconstructions. The full and pooled adult age-class models produced comparable estimates of natural survival and the vulnerability coefficients (Table 2). Similarly, results from the full age-class analysis [18] and pooled adult age-class analysis showed comparable trends in annual abundance (Table 3). The correlation between annual abundance estimates for the two approaches was *r* = 0.9739. The pooled adult age-class analysis estimated, on average, 17.4% more females annually than did the full age-class analysis. The full age-class analysis produced annual abundance estimates with an average CV of 31.01% compared with a CV = 31.61% for the pooled age-class analysis. Residual plots suggest comparable fits of the full and pooled adult age-class data to the population reconstruction models with scale parameters of 1.380 and 1.518, respectively (Fig. 1). These results are consistent with the general findings of the simulation study.

**Table 2:** Comparison of natural survival (*S*) and vulnerability coefficient (*c*) (SE) for reconstruction models using full age-class data and pooling of adult age-classes (2.5+ years).

Parameter	Full age-class data	Pooled adult age-class data
*S*	0.7220 (0.0172)	0.6953 (0.0197)
*c*_0.5_	0.0869 (0.0279)	0.0677 (0.0212)
*c*_1.5+_	0.1615 (0.0502)	0.1357 (0.0420)

**Table 3:** Estimates of female black-tailed deer abundance by year in Pierce County, Washington, USA, 1979–2002, based on pooled adult and full age-class population reconstructions.

Year	Full age-class data		Pooled age-class data	
	Annual abundance	95% confidence intervals	Annual abundance	95% confidence intervals
1979	3691.3	(1374.1, 6384.6)	4084.3	(1550.0, 7122.2)
1980	3150.7	(1234.1, 5569.5)	3644.4	(1443.8, 6474.2)
1981	2674.5	(1103.4, 4837.2)	3111.5	(1280.9, 5622.1)
1982	2558.3	(1082.0, 4679.0)	3211.9	(1343.3, 5844.7)
1983	2218.7	(954.9, 4090.2)	2718.8	(1147.4, 4967.7)
1984	1897.0	(784.6, 3434.7)	2279.4	(923.9, 4090.2)
1985	1604.5	(639.2, 2857.3)	1926.0	(751.7, 3399.3)
1986	1617.0	(643.8, 2878.8)	1997.9	(783.9, 3534.4)
1987	1531.8	(609.9, 2727.2)	1811.6	(706.0, 3195.3)
1988	1592.2	(635.5, 2837.8)	1896.7	(745.3, 3357.4)
1989	1566.0	(629.4, 2799.6)	1908.2	(752.5, 3383.1)
1990	1469.4	(587.3, 2620.5)	1815.5	(704.6, 3196.4)
1991	1530.9	(597.0, 2701.0)	1812.2	(680.5, 3145.9)
1992	1767.8	(674.5, 3089.8)	2095.4	(766.5, 3597.9)
1993	2001.9	(739.9, 3452.1)	2349.3	(833.4, 3982.6)
1994	2396.3	(876.7, 4114.6)	3345.4	(1184.7, 5667.4)
1995	2428.4	(897.8, 4188.1)	3218.3	(1159.7, 5491.3)
1996	2573.8	(960.0, 4455.5)	3034.8	(1102.8, 5196.2)
1997	3104.4	(1143.3, 5345.3)	3734.0	(1306.1, 6294.0)
1998	3142.8	(1173.0, 5441.8)	4016.3	(1438.3, 6835.4)
1999	2937.8	(1117.7, 5128.5)	3238.7	(1204.5, 5599.6)
2000	2536.7	(997.9, 4492.5)	3182.2	(1162.2, 5460.1)
2001	2107.2	(848.2, 3769.8)	2439.1	(896.5, 4196.4)
2002	1733.2	(700.8, 3106.8)	1980.1	(718.7, 3388.8)

## Discussion

The simulation studies demonstrate that big game reconstruction is feasible using as few as three age categories. There was enough age-structure information to perform a partial cohort analysis and estimate initial abundance of each recruitment class after pooling the adult age-at-harvest data. Furthermore, the reduction in precision was generally small (the average CV increased by 0.059) for the pooled adult age-class reconstructions. For ungulates such as white-tailed deer, mule deer (*Odocoileus hemionus*) and elk (*Cervus elaphus*), which are readily aged to young-of-the-year, subadults, and adults, the pooled adult age-class reconstruction method should provide useful abundance estimates. Tangible benefits to pooling adult age-class data would include reduced cost, particularly when data are collected at broad geographic scales, along with fewer logistical issues with estimating full age-class information each year. Additionally, the use of pooled age-class data means that other species, such as wild turkey (*Meleagris gallopavo*) which can sometimes be classified into broad age categories based on plumage and spur length, could be analyzed with statistical population reconstruction methods [20]. The age classes over which pooling occurs should depend on the biology of the species and when survival and harvest rates become homogeneous.

With sufficient auxiliary information, it is also possible to extend the pooling concept to only two age categories (e.g., young of year and adults) for applications such as small game species (e.g., greater sage-grouse [*Centrocercus urophasianus*], mourning doves [*Zenaida macroura*]) [21]. In extending the methods to small game species, additional auxiliary data were found to be essential in the reconstruction analysis [21]. The lack of cohort structure in the data required multiple sources of auxiliary demographic data before annual abundance could be reconstructed. Both catch-per-unit-effort and radiotelemetry information was necessary for model selection and estimability in a sage-grouse population reconstruction [21]. The statistical population reconstruction method offers a flexible framework that can incorporate a wide variety of auxiliary information such as telemetry data [17], [15], catch-effort [18], and abundance indices [18]. We strongly recommend the incorporation of auxiliary data in statistical population reconstruction, particularly in the case of pooled adult age classes where the cohort structure of the data is limited. Under such circumstances, full age-class and pooled age-class population reconstructions should perform better than even our simulation results suggest.

The statistical models used in population reconstruction are, at best, simplifications of reality. As such, the robustness of the reconstruction should be evaluated. One approach is to use multimodal inference techniques of [22]. We further recommend using data deletion techniques to determine the stability of the estimated abundance trend. The procedure deletes historical data one year at a time, and the abundance trends are reconstructed each time. If the results are sensitive to the removal of one or a few years of data, the original reconstruction should be viewed with heightened concern. Conversely, robustness of the reconstruction results to the amount of historical data removed should provide additional reassurance in the final results.

Given flexibility in model construction and incorporation of auxiliary information, the use of statistical population reconstruction could be applied to situations where (1) historically only pooled adult age-class data are available; (2) collection of full age-class data are costly; (3) logistical constraints dictate the collection of pooled age-class data; (4) animals can only be reliably classified into broad age categories; and (5) assignment of full age-class data are not possible due to errors in aging (e.g., cementum annuli; [5]). With increasingly tight management budgets, it is more difficult to continue the collection of data that are expensive, such as cementum annuli counts. The reconstruction methods described herein offer one alternative technique for demographic assessment that might reduce costs without a substantial loss in associated information or precision. Ultimately managers should consider the intended purpose, the necessary accuracy and precision of demographic values, and feasibility of data collection when deciding whether to pool age-class data or not.

### Management Implications

Population reconstruction using pooled adult age-class data can provide a cost-effective supplement to existing inventory methods, and in some cases, could provide the primary method of inventorying hunted game populations over large geographic areas. Tooth eruption and wear data are relatively easy and inexpensive to collect from harvested ungulates when compared with other methods, and in most cases, can be used to accurately age individuals to the young-of-year, subadults, and adults (2.5+ years). Our analysis suggests reliable population trends can be reconstructed with only a small loss of precision and without the need for expensive tooth extraction and cementum annuli analyses. Aging by tooth eruption and wear is already commonly used by many wildlife agencies, and this paper suggests a useful means of analyzing this often collected and neglected demographic data. When implementing population reconstruction as a management tool, auxiliary studies should be a required part of any management plan in order to provide auxiliary data for the demographic analysis.

### Availability and Future Directions

The maximum likelihood models for the full age-class and pooled adult age-class population reconstruction were constructed and numerically analyzed using the freeware Program USER 4.0 (User Specified Estimation Routine) available from the University of Washington at: http://www.cbr.washington.edu/paramest/user/. Program USER provides an iterative environment for investigators to construct multinomial and product multinomial likelihood functions. Ongoing research is investigating the utility of random effect versions of the existing population reconstruction models and the use of AD Model Builder (http://otter-rsch.com/admodel.htm) to numerically solve the likelihoods.
